# Molecular Alterations Associated with Histologically Overt Stromal Response in Patients with Prostate Cancer

**DOI:** 10.3390/ijms25168913

**Published:** 2024-08-16

**Authors:** Mutlay Sayan, Yetkin Tuac, Mahmut Akgul, Samet Kucukcolak, Elza Tjio, Dilara Akbulut, Luke W. Chen, David D. Yang, Shalini Moningi, Jonathan E. Leeman, Peter F. Orio, Paul L. Nguyen, Anthony V. D’Amico, Cagdas Aktan

**Affiliations:** 1 Department of Radiation Oncology, Brigham and Women’s Hospital and Dana Farber Cancer Institute, Harvard Medical School, Boston, MA 02115, USA; 2 Department of Statistics, Ankara University, Ankara 06100, Türkiye; 3 Department of Pathology and Laboratory Medicine, Albany Medical Center, Albany, NY 12208, USA; 4 Department of Pathology and Laboratory Medicine, Rutgers University, New Brunswick, NJ 07102, USA; 5 Histopathology Department, Harrogate District Hospital, Harrogate HG2 7SX, UK; 6 Laboratory of Pathology, Center for Cancer Research, National Institutes of Health, Bethesda, MD 20892, USA; 7 Department of Medical Biology, Faculty of Medicine, Bandirma Onyedi Eylul University, Balikesir 10250, Türkiye

**Keywords:** prostate cancer, HOST-response, genomic instability, tumor microenvironment

## Abstract

Prostate cancer has substantial heterogeneity in clinical outcomes and therapeutic responses, posing challenges in predicting disease progression and tailoring treatment strategies. Recent studies have highlighted the potential prognostic value of evaluating the tumor microenvironment, including the presence of a histologically overt stromal response (HOST-response) characterized by peri-glandular stromal changes and architectural distortions. This retrospective study examined patient records from The Cancer Genome Atlas database to identify genomic alterations associated with the HOST-response in prostate cancer. Among 348 patients who underwent radical prostatectomy, 160 (45.98%) were identified as having a HOST-response. A gene expression analysis revealed 1263 genes with significantly higher expression in patients with a HOST-response. A protein–protein interaction network analysis identified seven hub genes (*KIF2C, CENPA, CDC20, UBE2C, ESPL1, KIF23,* and *PLK1*) highly interconnected in the network. A functional enrichment analysis revealed alterations in the cell division, cytoskeletal organization, cytokinesis, and interleukin-16 signaling pathways in patients with a HOST-response, suggesting dysregulated proliferation and inflammation. The distinct molecular signature associated with the HOST-response provides insights into the tumor–stroma interactions driving adverse outcomes and potential targets for tailored therapeutic interventions in this subset of patients with prostate cancer.

## 1. Introduction

Prostate cancer is a highly heterogeneous disease, exhibiting substantial variability in clinical outcomes and therapeutic responses among patients [1,2,3,4]. This heterogeneity poses significant challenges in predicting disease progression and tailoring treatment strategies effectively. Recent studies have highlighted the potential prognostic value of evaluating the tumor microenvironment in prostate cancer [5]. One notable feature observed in a subset of prostate cancer cases is the presence of a histologically overt stromal response (HOST-response), characterized by peri-glandular stromal changes, desmoplasia-like alterations, and architectural distortions in the prostate cancer glands [5,6,7,8]. Our previous study demonstrated that the presence of a HOST-response is significantly associated with reduced progression-free survival (PFS) in patients treated with radical prostatectomy, even after adjusting for well-established prognostic factors [9], with PFS being defined as the duration from the completion of radical prostatectomy to the time of cancer progression—characterized by biochemical recurrence, locoregional recurrence, or distant metastasis—or death from any cause, whichever occurred first.

The tumor microenvironment plays a crucial role in cancer development and progression, with complex interactions between cancer cells and the surrounding stromal components, including fibroblasts, immune cells, and the extracellular matrix [10,11,12]. These interactions can influence various aspects of tumor behavior, such as proliferation, invasion, metastasis, and therapeutic resistance [12,13,14,15,16]. Genomic alterations in cancer cells can contribute to the remodeling of the tumor microenvironment, leading to the recruitment and activation of stromal cells as well as the deposition of extracellular matrix components [17,18,19,20]. Conversely, the tumor microenvironment can exert selective pressures on cancer cells, driving the acquisition of additional genomic alterations that confer a survival advantage [17,18,19,20,21,22].

While the clinical implications of a HOST-response have been established, the underlying molecular mechanisms driving this stromal response remain poorly understood. Elucidating the genomic alterations associated with a HOST-response could provide valuable insights into the biological processes involved in tumor–stroma interactions and their impact on disease progression. In this study, we sought to identify and characterize the molecular alterations associated with the presence of a HOST-response in patients with prostate cancer.

## 2. Results

### 2.1. Study Population and Gene Expression Analysis

Among the 342 patients who met the study inclusion criteria, 160 (45.98%) were identified as having a HOST-response. This is consistent with our previous findings [9], where a similar prevalence of HOST-response was observed. A total of 19,809 genes were analyzed, and 932 of these genes demonstrated significantly higher expression levels in patients with a HOST-response compared to those without.

### 2.2. Protein–Protein Interaction Network and Hub Genes

**Figure 1:**
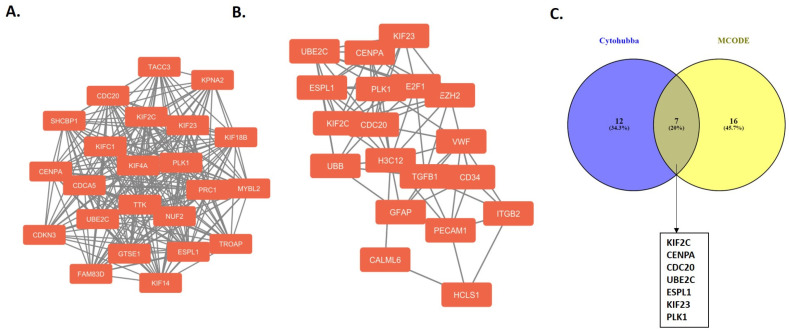
A protein–protein interaction network analysis of differentially expressed genes in patients with a HOST-response. The nodes represent proteins, and the edges represent the predicted functional associations. (**A**) The most significant module was analyzed using the MCODE application in the Cytoscape software v3.10.1. (**B**) The hub genes were analyzed using the Cytohubba application in the Cytoscape software. (**C**) A Venn diagram illustrating the intersecting genes identified through Cytohubba and MCODE analyses.

The constructed protein–protein interaction (PPI) network comprised 879 nodes (proteins) and 2542 edges (interactions between proteins). The enrichment analysis of the PPI network yielded a *p*-value of 1.0 × 10^−16^, indicating a highly significant and non-random interaction network. In addition, an interaction network was constructed for the differentially expressed genes (DEGs) in patients with a HOST-response and their neighboring genes. According to the Molecular Complex Detection (MCODE) score rankings, the highest-scoring module was selected for detailed visualization (Figure 1A; MCODE score = 21.818). As shown in Figure 1A, the module consists of 23 nodes and 240 edges, illustrating the interactions among proteins in patients with a HOST-response. Similarly, the top 25 hub genes among the DEGs were identified using the Cytohubba tool. These hub genes were identified using degree and closeness algorithms, with the results illustrated in Figure 1B. Additionally, the results from both the MCODE and Cytohubba analyses were intersected using a Venn diagram, identifying seven overlapping hub genes (*KIF2C, CENPA, CDC20, UBE2C, ESPL1, KIF23,* and *PLK1*) in patients with a HOST-response (Figure 1C). These hub genes were found to be significant in both analyses, as shown in Figure 1C, which illustrates the common intersecting genes. Among these hub genes, *ESPL1* had a missense mutation (I949V) in patients with a HOST-response.

**Figure 2:**
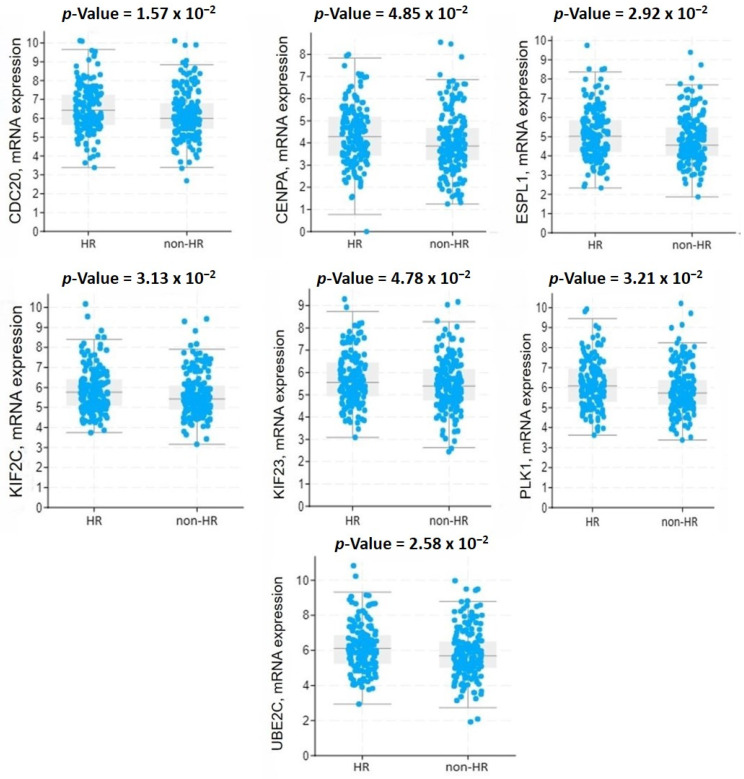
The mRNA expression level of the hub genes. Abbreviations: HR, histologically overt stromal response (HOST-response); non-HR, non-HOST-response.

To evaluate the expression levels of the hub genes, an mRNA expression analysis was conducted. As shown in Figure 2, the expression levels of *KIF2C, CENPA, CDC20, UBE2C, ESPL1, KIF23,* and *PLK1* were significantly higher in the tumor tissues of patients with a HOST-response compared to those without a HOST-response.

### 2.3. Functional Enrichment Analysis

**Figure 3:**
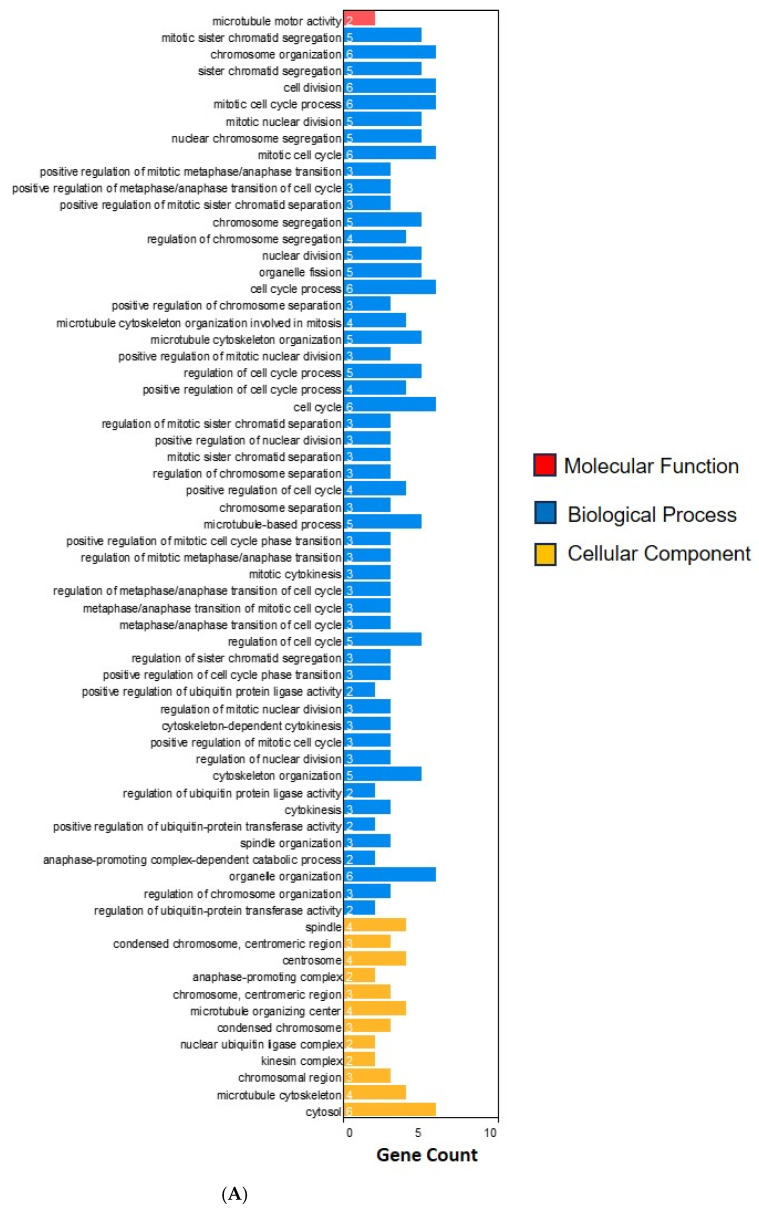

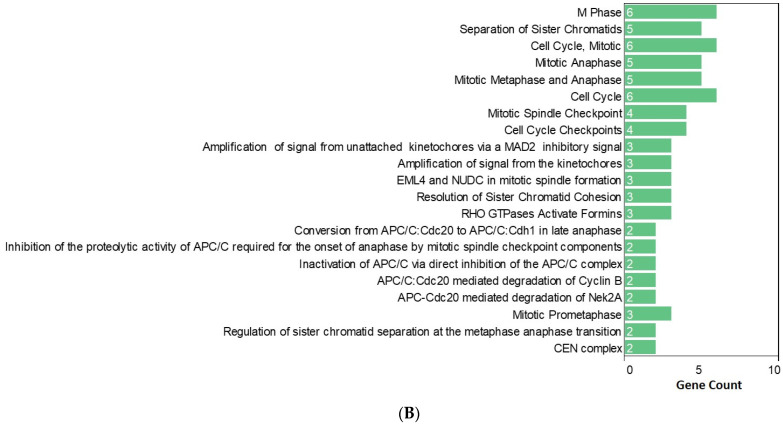
An enrichment analysis of differentially expressed genes. (**A**) A Gene Ontology enrichment analysis. (**B**) A pathway enrichment analysis using the Reactome, WikiPathways (WP), and CORUM databases. The x-axis represents the number of enriched differentially expressed genes, and the y-axis shows the enriched GO terms (**A**) and pathways from each database (**B**).

The functional enrichment results are shown in Figure 3. The Gene Ontology (GO) biological process analysis revealed alterations in several key biological processes associated with the prostate cancer pathology in patients with a HOST-response. Notably, there was increased activity in cell division (GO:0051301), cytoskeleton organization (GO:0007010), and cytokinesis (GO:0000910), suggesting that these biological processes may play significant roles in the disease progression in patients with a HOST-response.

The GO molecular function analysis aimed to define the molecular activities of specific proteins in patients with a HOST-response. The analysis identified several critical functions, including interleukin-16 (IL-16) receptor activity (GO:0042012) and IL-16 binding (GO:0042011).

The GO cellular component analysis focused on defining the molecular structures of specific cellular components in patients with a HOST-response. The analysis indicated altered activities in microtubule motor activity (GO:0003777). 

The Reactome, WP, and CORUM pathway enrichment analyses identified that the genes in patients with a HOST-response were primarily enriched in pathways related to the cell cycle checkpoints (REAC:R-HSA-69620), the amplification of a signal from unattached kinetochores via a MAD2 inhibitory signal (REAC:R-HSA-141444), the APC/C:Cdc20-mediated degradation of Cyclin B (REAC:R-HSA-174048), the APC-Cdc20-mediated degradation of Nek2A (REAC:R-HSA-179409), the regulation of sister chromatid separation at the metaphase anaphase transition (WP:WP4240), and the CEN complex (CORUM:929).

### 2.4. The Prognostic Value of the Hub Genes

**Figure 4:**
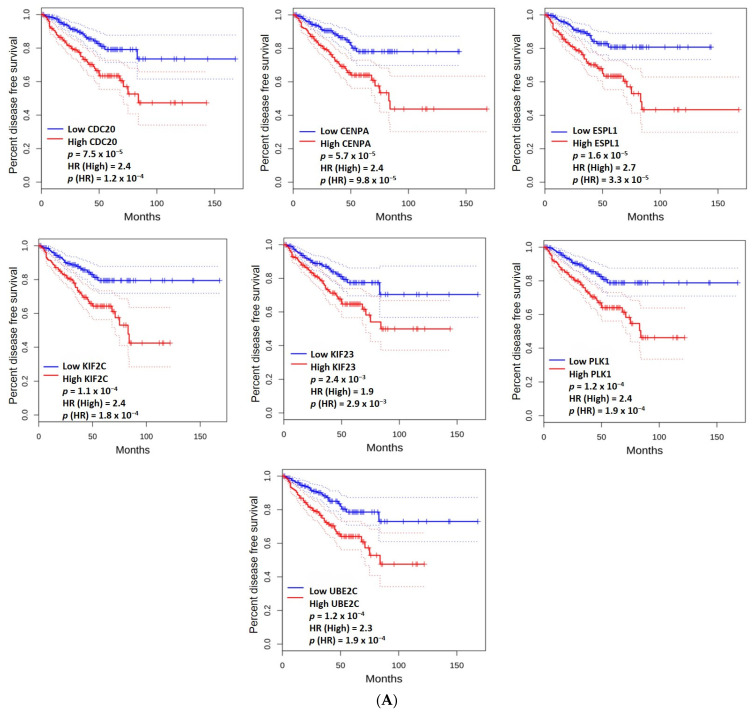

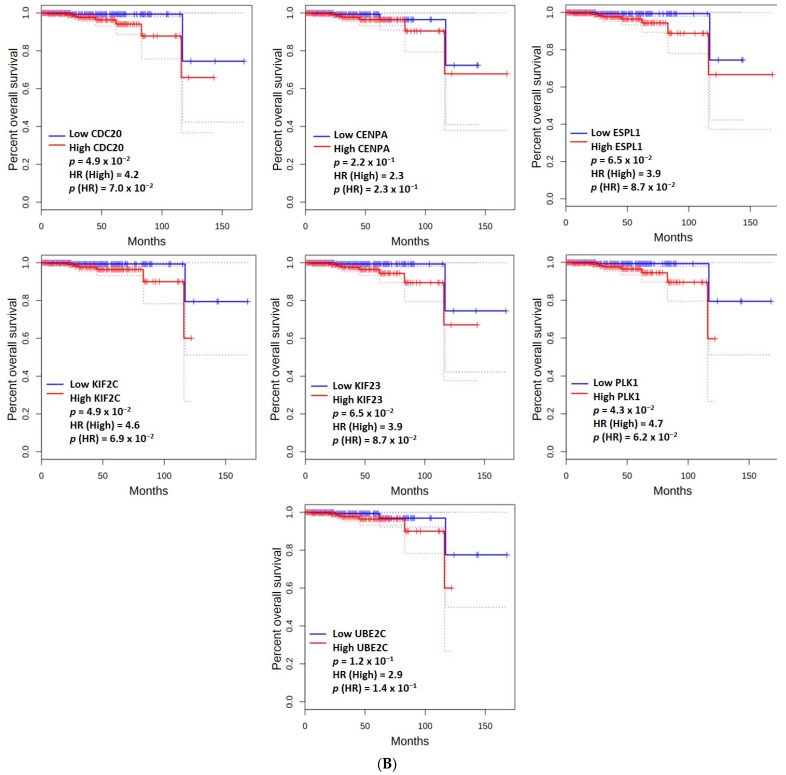
The disease-free survival (**A**) and overall survival (**B**) based on the gene expression levels of the hub genes. The median is selected as a threshold for separating high-expression and low-expression cohorts. The red and blue blocks represent higher and lower risks, respectively. The solid line represents the survival curve, and the dotted line represents the 95% confidence interval. The HR (Hazard Ratio) is a measure of the relative risk of an event occurring in the high-expression group compared to the low-expression group. The *p*(HR) value is associated with the HR and indicates whether the observed HR is statistically significant. If *p*(HR) < 0.05, the HR is considered statistically significant, suggesting that the difference in risk between the high- and low-expression groups is unlikely to be due to chance alone. A *p*-value < 0.05 (log-rank test) was considered significant.

High mRNA expression levels of *KIF2C* (*p* = 1.1 × 10^−4^), *CENPA* (*p* = 5.7 × 10^−5^), *CDC20* (*p* = 7.5 × 10^−5^), *UBE2C* (*p* = 1.2 × 10^−4^), *ESPL1* (*p* = 1.6 × 10^−5^), *KIF23* (*p* = 2.4 × 10^−3^), and *PLK1* (*p* = 1.2 × 10^−4^) were associated with a shorter disease-free survival (Figure 4A). High mRNA expression levels of *KIF2C* (*p* = 4.9 × 10^−2^), CDC20 (*p* = 4.9 × 10^−2^), and *PLK1* (*p* = 4.3 × 10^−2^) were associated with lower overall survival (Figure 4B).

## 3. Discussion

The findings from this study provide important insights into the molecular underpinnings of the HOST-response in prostate cancer and its association with adverse clinical outcomes, as reported in our previous work [9]. Through comprehensive genomic analyses, we identified a distinct molecular signature characterized by dysregulated pathways and hub genes that may drive the formation and propagation of the reactive tumor stroma. The clinical significance of these findings is the identification of potential therapeutic targets and biomarkers that could improve outcomes for patients with prostate cancer exhibiting a HOST-response.

A key observation from our study is the significant enrichment of cell cycle-related biological processes among the DEGs in HOST-response-positive tumors, indicating a heightened state of cellular proliferation. This is evidenced by the upregulation of genes such as *KIF2C*, *CENPA*, *CDC20*, *UBE2C*, *ESPL1*, *KIF23*, and *PLK1*, which are critical regulators of cell cycle progression. These genes play essential roles in various aspects of mitosis, chromosome segregation, and spindle formation, highlighting their importance in maintaining genomic stability and proper cell division [23]. The upregulation of these genes suggests an increased proliferative capacity within the tumor microenvironment, aligning with the well-established role of the reactive stroma in promoting tumor growth and progression. Our analysis revealed that high mRNA expression levels of *KIF2C*, *CDC20*, and *PLK1* are significantly associated with a lower OS, while high mRNA expression levels of *KIF2C*, *CENPA*, *CDC20*, *UBE2C*, *ESPL1*, *KIF23*, and *PLK1* are associated with shorter DFS. This further highlights the potential prognostic value of these genes in prostate cancer. For instance, *KIF2C* is crucial for chromosome segregation and spindle assembly during mitosis, and its overexpression is associated with poor prognosis in several cancers, including breast and lung cancers [24,25,26,27]. Similarly, *CENPA* is essential for kinetochore assembly and chromosome segregation, with its overexpression leading to chromosomal instability, a hallmark of cancer [28,29,30]. *CDC20*, an activator of the anaphase-promoting complex/cyclosome, regulates the transition from metaphase to anaphase, and its aberrant expression is linked to various cancers [31,32]. *UBE2C*, involved in the ubiquitin–proteasome pathway, regulates the degradation of cell cycle proteins, and its overexpression is associated with enhanced cell proliferation and poor prognosis in multiple cancers [33,34,35]. *ESPL1*, crucial for sister chromatid separation during anaphase, and *KIF23*, involved in cytokinesis, both contribute to increased cell proliferation and tumorigenesis when overexpressed [36,37]. Lastly, *PLK1*, a key regulator of various stages of mitosis, is commonly overexpressed in many cancers and is associated with poor prognosis and increased tumor cell proliferation [38,39]. Collectively, these findings highlight the critical role of cell cycle-related genes in the aggressive behavior of HOST-response-positive tumors and emphasize the importance of further research into their potential as biomarkers and therapeutic targets.

The upregulation of these hub genes in HOST-response-positive tumors suggests that the reactive stroma may create a microenvironment conducive to increased cell division and genomic instability [40]. This is supported by the enrichment of pathways related to cell cycle checkpoints, kinetochore signaling, and chromatid separation, such as the APC/C:Cdc20-mediated degradation of Cyclin B and the regulation of sister chromatid separation at the metaphase–anaphase transition [41]. The dysregulation of these pathways can lead to unchecked cell proliferation and the accumulation of genetic mutations, driving tumor progression and resistance to therapy. For example, the APC/C:Cdc20 pathway is critical for the proper segregation of chromosomes during cell division, and its dysregulation can result in aneuploidy and chromosomal instability, which are common features of aggressive cancers [42]. Furthermore, the identification of the CEN complex as an enriched pathway is particularly noteworthy given the role of centromere proteins in chromosome segregation and mitotic progression [43,44,45]. This finding, along with the upregulation of genes like *CENPA*, suggests that chromosomal instability may be a feature of HOST-response tumors, further emphasizing the complex interplay between the tumor microenvironment and genetic instability in cancer progression.

Interestingly, our analysis revealed alterations in pathways related to IL-16 receptor activity and binding, suggesting a potential role for inflammatory signaling in the HOST-response. IL-16 is a pro-inflammatory cytokine that recruits and activates immune cells such as CD4+ T cells, monocytes, and eosinophils, which can contribute to a tumor-promoting microenvironment [46,47,48]. The upregulation of IL-16 receptor activity in HOST-response-positive tumors indicates chronic inflammation, a known driver of cancer progression [49]. This chronic inflammatory state can enhance tumor growth, angiogenesis, and metastasis by interacting with other inflammatory pathways like NF-κB and STAT3 [50,51]. Additionally, IL-16 signaling may influence cancer-associated fibroblasts and extracellular matrix remodeling, further facilitating tumor invasion and metastasis [52]. These findings highlight the complex interplay between inflammation and cancer, providing potential targets for therapeutic intervention to disrupt the pro-tumorigenic inflammatory microenvironment in prostate cancer.

While our study provides valuable insights into the molecular landscape of the HOST-response, there are some limitations that warrant further investigation. First, our analyses were based on bulk tumor samples, which may obscure the heterogeneity within the tumor microenvironment. Future studies employing single-cell transcriptomics or spatial transcriptomics could provide a more comprehensive understanding of the cellular interactions and spatial organization of the reactive stroma. Second, although 160 patients with a HOST-response were included in this analysis, a larger and more diverse sample size might yield more comprehensive and generalizable results. Finally, functional validation experiments, such as in vitro co-culture systems or in vivo models, could help clarify the causal relationships between the identified molecular alterations and the formation of the HOST-response in patients with prostate cancer.

Despite these limitations, our findings have important clinical implications. The identification of a distinct molecular signature associated with the HOST-response provides a potential avenue for developing targeted therapeutic strategies. For instance, inhibitors of the key pathways or hub genes identified in this study could be explored as adjuvant therapies to disrupt the formation of the reactive stroma and potentially improve treatment outcomes. Furthermore, the molecular signature could serve as a biomarker for patient stratification, enabling the identification of individuals who may benefit from such targeted interventions. Additionally, future research could explore the potential of liquid biopsies, such as urine or blood samples, to detect collagen-related biomarkers associated with the HOST-response. Recent studies have shown promise in using urine-based collagen signatures for predicting clinically significant prostate cancer, suggesting that non-invasive fluid-based tests could complement tissue analysis in risk stratification and personalized treatment decisions [53].

## 4. Materials and Methods

### 4.1. Study Population and Pathological Assessment

In this retrospective study, we examined patient records from The Cancer Genome Atlas (TCGA) database. Patients with a confirmed diagnosis of prostate cancer who had undergone radical prostatectomy without neoadjuvant therapy and had digital slides stained with hematoxylin and eosin were included in this study.

A team of three pathologists specializing in genitourinary disease (identified as MA, ET, and SK) evaluated the radical prostatectomy specimens to identify cases with a HOST-response. Each pathologist reviewed every case independently to ensure thorough analysis, and in cases of disagreement among pathologists, the majority view was adopted if consensus could not be achieved. As reported in our previous study [9], a HOST-response describes the peri-glandular stromal changes leading to desmoplasia-like alterations in the stroma and structural changes in the prostate cancer glands, often resulting in angulation and retraction artifacts [5,6,9]. Tumors having more than 50% reactive stroma relative to the total tumor area were classified as stromal grade 3, where the reproducibility of identifying a HOST-response is the highest [6,9]. Therefore, our definition of a HOST-response in this study refers to grade 3 stromogenic changes. Labels of the scanned HE whole slide images were compared with the label of the aliquot used for sequencing.

### 4.2. Gene Expression Analysis

The gene expression profiling dataset from the TCGA database was used to examine the expression levels of mRNAs. DNA extraction and sequencing were conducted on both fresh-frozen and formalin-fixed paraffin-embedded tissues using high-throughput next-generation sequencing technologies [1]. The RNASeq V2 data, processed and normalized using RNA-Seq by Expectation-Maximization, provided the basis for identifying DEGs [1]. DEGs were identified in patients with and without a HOST-response by meeting the criteria of an absolute fold change (|FC|) greater than 1 or less than −1, along with a *p*-value below 0.05 [54,55].

### 4.3. Construction of Protein–Protein Interaction Network and Identification of Hub Genes

The Search Tool for the Retrieval of Interacting Genes v12.0 (https://string-db.org/ (accessed on 29 July 2024)) was utilized to construct a PPI network [56]. Cytoscape software v3.10.2 was used to visualize the constructed PPI network [57]. MCODE tool v2.0.3 in Cytoscape was used to identify significant genes in the subnetwork, with parameters set to a K-score of 2, a degree cutoff of 2, a node cutoff of 0.2, and a maximum depth of 100 [58]. To identify the most intersected key genes and modules, the Cytohubba tool v0.1 was applied in Cytoscape, and PPI-MCODE modules were merged [59]. As a result, hub genes—highly interconnected genes within the PPI network—were identified by selecting those deemed significant by both the MCODE and Cytohubba tools.

### 4.4. Functional Enrichment Analysis

Functional enrichment analysis was conducted using GO analysis to determine the biological significance of the hub genes. Gene functionalities are typically classified into three categories: biological processes, molecular functions, and cellular components. Annotation and pathway enrichment analysis of DEGs were conducted using the comprehensive gProfiler web tool (https://biit.cs.ut.ee/gprofiler/gost (accessed on 29 July 2024)) [60] and the Reactome, WP (WikiPathways), and CORUM databases [61].

### 4.5. The Prognostic Value of the Hub Genes

OS and DFS analyses based on the gene expression levels of the hub genes were performed using the log-rank test on GEPIA (http://gepia.cancer-pku.cn/ (accessed on 29 July 2024)). The Cox proportional hazard ratio (HR) and the 95% confidence intervals (CIs) for the survival plots were provided.

## 5. Conclusions

This study reveals a complex molecular landscape associated with the HOST-response in prostate cancer, characterized by alterations in cell cycle regulation, chromosomal stability, and cytoskeletal organization. These findings not only provide mechanistic insights into the adverse clinical outcomes associated with the HOST-response but also pave the way for the development of novel therapeutic strategies and biomarkers tailored to this unique tumor microenvironment. Further research, including the functional validation of the identified hub genes and pathways, will be essential to fully elucidate the mechanisms underlying the HOST-response and its impact on prostate cancer progression and treatment response.

## Data Availability

The original contributions presented in this study are included in this article. Further inquiries can be directed to the corresponding author.
